# Effect of Pore Structure Parameters on Thermal Insulation Performance of Porous Ceramics Fabricated by Material Jetting

**DOI:** 10.3390/ma19122667

**Published:** 2026-06-21

**Authors:** Qintao Shen, Peng Wang, Chunan Song, Chao Ding, Yapeng Ning, Viboon Saetang, Mengji Shen, Yaxuan Wei, Jiying Wang, Renquan Ji, Xin Yang, Huan Qi

**Affiliations:** 1 Zhejiang Key Laboratory of Aerospace Metallic Materials, Hangzhou City University, Hangzhou 310015, China; sqt@hzcu.edu.cn (Q.S.); wangp@hzcu.edu.cn (P.W.); dingchao@hzcu.edu.cn (C.D.); ningyp@hzcu.edu.cn (Y.N.); 2250306020@hzcu.edu.cn (M.S.); huanqi@hzcu.edu.cn (H.Q.); 2 Zhejiang-Thailand International Joint Laboratory on New Materials Digital Design and Processing Technology, Hangzhou City University, Hangzhou 310015, China; viboon.tan@kmutt.ac.th; 3 School of Engineering, Hangzhou City University, Hangzhou 310015, China; 4 School of Mechanical Engineering, Zhejiang University, Hangzhou 310030, China; 5 School of Materials Science and Engineering, Zhejiang University, Hangzhou 310030, China; 13227788157@163.com; 6 Department of Production Engineering, Faculty of Engineering, King Mongkut’s University of Technology Thonburi, Bangkok 10140, Thailand; 7 Zhejiang Metallurgical Research Institute Co., Ltd., Hangzhou 310011, China; weiyx19@tsinghua.org.cn (Y.W.); wangjiying@hzsteel.com (J.W.)

**Keywords:** Material Jetting, porous ceramics, pore structure parameters, thermal insulation performance, zirconia

## Abstract

Porous ceramics have shown great application potential in aerospace, electronics, and lithium-ion battery thermal management due to their low density, high specific strength, and excellent thermal insulation. Material Jetting (MJ), a high-precision 3D printing technology, enables the fabrication of porous ceramics with tailored pore structures, but the synergistic effects of pore structure parameters (configuration, porosity, and number of periods) on their thermal insulation performance remain insufficiently explored. This study systematically investigates the thermal insulation behavior of zirconia porous ceramics fabricated by MJ through experimental tests and numerical simulations. Three typical lattice configurations (Octet, Schwarz, and Gyroid) were selected, and samples with varying porosities (40%, 50%, 60%) and numbers of periods (1, 2, 3) were prepared. The results indicate that the Octet configuration (60% porosity, 3 periods) exhibits the optimal thermal insulation performance, with a minimum cold-end temperature of 58.5 °C (experiment) and 59.21 °C (simulation), attributed to its strut-based structure that forms a more tortuous heat conduction path. For the Gyroid configuration, thermal insulation performance improves with increasing porosity (reducing solid conduction dominance under non-forced convection) and decreases with decreasing number of periods (due to inhomogeneous pore distribution extending heat transfer paths). Notably, the trend of porosity affecting thermal insulation is opposite to that of compressive performance. Numerical simulation results are consistent with experimental data in both values and trends, verifying the reliability of the model. This work clarifies the key factors regulating the thermal insulation of MJ-fabricated porous ceramics and provides practical structural design guidelines for applications such as lithium-ion battery thermal runaway management.

## 1. Introduction

Advanced materials development is a cornerstone of technological progress, driving breakthroughs in aerospace, biomedical engineering, and other fields [1,2,3,4,5,6,7]. Porous materials, distinguished by their unique pore structures, exhibit low density, high specific surface area, superior energy absorption, and tunable permeability, making them essential in many critical industries [8,9]. In biomedicine, additively manufactured porous metals act as bone scaffolds, supporting cell infiltration and osseointegration [10,11,12]. In aerospace and automotive engineering, lightweight porous structures improve fuel efficiency and structural safety [13,14]. For energy and environmental applications, they enable efficient gas separation, water purification, and catalysis. Recently, porous ceramics have attracted extensive attention for their low density, high specific strength, excellent thermal insulation, and corrosion resistance in aerospace, electronics, and energy storage [15,16]. The combination of porous materials and advanced additive manufacturing technologies (e.g., LB-PBF, EB-PBF) overcomes the limitations of traditional fabrication, enabling precise microscale pore design and customized mechanical and functional properties [17,18,19,20,21,22]. Among 3D-printing methods, Material Jetting (MJ) features high precision and fine resolution, making it particularly suitable for fabricating porous ceramics with complex lattice structures [23,24,25].

The thermal insulation performance of porous ceramics is predominantly determined by their pore structure parameters, including configuration, porosity, and number of periods [26]. Lattice structures, as a type of ordered porous structure, are classified into strut-based and surface-based categories [27,28]. Strut-based lattice structures (e.g., Octet truss) exhibit excellent mechanical properties due to their symmetric design, while surface-based structures, particularly Triply Periodic Minimal Surfaces (TPMS) such as Schwarz and Gyroid, are renowned for their continuous and smooth surfaces that can reduce stress concentration [29,30]. Lithium-ion battery thermal runaway management is a critical application scenario for porous ceramic thermal insulation materials. When the temperature of lithium-ion batteries exceeds 200 °C, thermal runaway is prone to occur, and rapid heat diffusion can lead to catastrophic failures of battery modules. Therefore, developing porous ceramics with superior thermal insulation performance for battery thermal management is of great practical significance.

Recent studies have begun to investigate the thermal transport behavior of additively manufactured porous ceramics and TPMS architectures. Yang et al. demonstrated that porosity and lattice number significantly affect the thermal insulation performance of DLP-fabricated zirconia TPMS ceramics [31]. DiReda et al. further showed that TPMS topology plays an important role in determining the thermal response of additively manufactured ceramic structures [32]. These findings highlight the strong dependence of thermal insulation performance on lattice architecture and geometric parameters. While MJ technology enables precise fabrication of porous ceramics with tailored lattice structures and thus holds immense promise for high-performance thermal insulation applications, existing research on such ceramics remains far from comprehensive and targeted. Although several recent studies have explored the thermal transport behavior of porous ceramic lattice and TPMS structures, existing research has predominantly focused on their mechanical performance, such as compressive strength and energy absorption capacity, with scarce systematic investigations into how core pore structure parameters-configuration, porosity, and number of periods-synergistically modulate thermal insulation performance [33,34,35]. Moreover, the intrinsic heat transfer mechanisms underlying strut-based and surface-based lattice structures, which dictate their thermal insulation behavior under non-forced convection conditions (the typical working state for practical thermal management scenarios), have not yet been clearly elucidated. Additionally, there is a critical lack of reliable, application-oriented structural design guidelines for MJ-fabricated porous ceramics tailored to thermal insulation demands, especially for urgent engineering scenarios like lithium-ion battery thermal runaway management [36]. Specifically, the specific regulatory mechanisms of configuration, porosity, and period number on heat transfer in MJ-fabricated porous ceramics remain unclear, and no actionable structure-design criteria for thermal insulation applications have been established. This vital knowledge gap not only obscures the key factors governing thermal insulation performance but also severely impedes the rational structural design and practical industrial application of these advanced porous ceramics in thermal management fields. Most existing studies on lattice and TPMS structures have primarily focused on metallic or conventionally fabricated ceramic systems, often employing fabrication routes [37,38,39], and conducted under simplified or inconsistent boundary conditions. In contrast, investigations targeting MJ-fabricated zirconia porous ceramics remain limited, particularly in terms of systematic comparisons across structural parameters within a unified experimental–numerical framework. These limitations highlight the need for a consistent and process-specific evaluation of structure-thermal performance relationships in MJ-fabricated porous ceramics.

To address these research gaps, this study investigates the effects of pore structure parameters (configuration, porosity, period number) on the thermal insulation performance of MJ-fabricated zirconia porous ceramics. We prepared samples of three typical lattice configurations (Octet, Schwarz, Gyroid) with varied porosities (40%, 50%, 60%) and period numbers (1, 2, 3), and combined experimental tests with ANSYS Workbench-based numerical simulations to analyze thermal insulation behavior and heat transfer mechanisms. This work seeks to clarify the regulatory effects of the parameters, verify experimental-simulation consistency, and provide theoretical and practical references for the structural design of MJ-fabricated porous ceramics in thermal insulation applications.

## 2. Materials and Methods

### 2.1. Selection of Porous Ceramic Structures

Since the concept of porous structures was proposed by Gibson and Ashby [40], it has evolved from regular to irregular, single-material to composite, and simple geometric to topological structures. As a type of ordered porous structure, lattice structures offer numerous advantages such as light weight, high strength, and efficient heat dissipation, and all porous structures described in this study adopt lattice structures.

**Figure 1:**
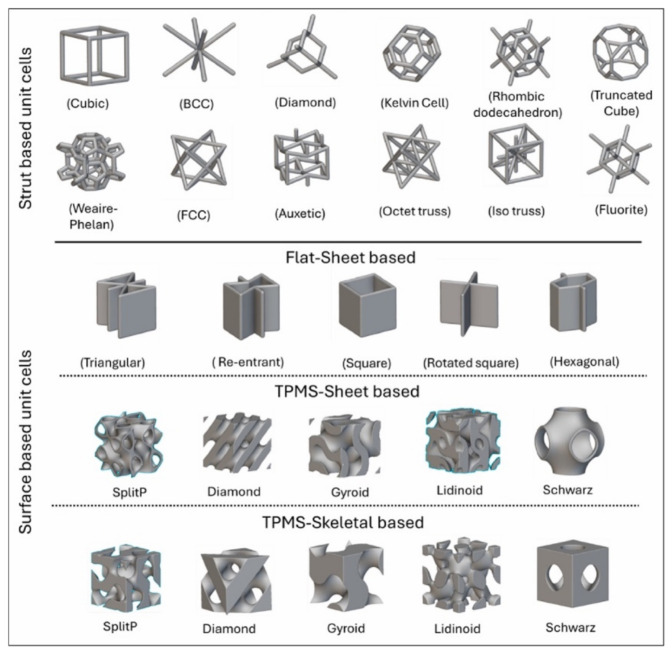
Common porous structures in 3D printing [41].

As shown in Figure 1, common lattice structures in 3D printing are classified into strut-based and surface-based categories according to their structural characteristics. Strut-based lattice structures have nodes located at the vertices or edges (and sometimes inside) of unit cells, connected by slender straight rod elements. In contrast, surface-based lattice structures are composed of surface layers assembled into three-dimensional structures, including flat sheets and TPMS [41].

**Figure 2:**
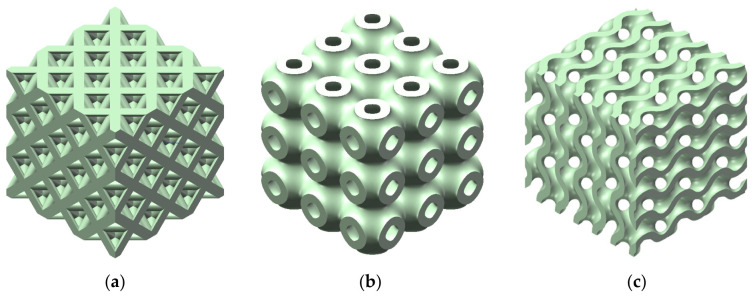
Three types of porous structure configurations used for analysis: (**a**) Octet (**b**) Schwarz (**c**) Gyroid.

To analyze the effect of different pore structures on the thermal insulation performance of MJ-fabricated porous ceramics, it is necessary to select appropriate models considering the typicality and forming characteristics of various structures. Three configurations—Octet, Schwarz, and Gyroid—were chosen for this study. As shown in Figure 2a, the Octet configuration is a spatial lattice structure composed of interconnected octahedral and tetrahedral units. Owing to its high structural symmetry and low stress concentration, it exhibits excellent specific strength, stiffness, and energy absorption capacity, making it highly advantageous in load-bearing and energy-absorbing applications. The Schwarz and Gyroid configurations, as shown in Figure 2b,c, are typical TPMS structures with smooth, continuous curved surfaces that can effectively reduce stress concentration and improve stress distribution. Additionally, the continuous nature of their curved surfaces can significantly reduce heat transfer paths and efficiency, thereby enhancing thermal insulation performance.

Thus, lattice structures are categorized into strut-based and surface-based types by their structural characteristics, and Octet, Schwarz and Gyroid configurations were selected for this study based on rigorous practical considerations. As the typical representative of strut-based lattices, the Octet features high structural symmetry, while Schwarz and Gyroid are classic TPMS surface-based lattices with continuous and smooth curved surfaces; the distinct structural features of the three configurations allow for a systematic comparison of thermal insulation behaviors between different lattice types. Meanwhile, all three structures are compatible with the high-precision forming characteristics of MJ technology, with no geometric features that would lead to forming defects, and can be fabricated with wall thicknesses meeting MJ’s processing requirements (≥400 μm). More importantly, their structural differences are highly conducive to exploring the intrinsic correlation between lattice morphology and heat transfer mechanisms, the core focus of this thermal insulation research. Thus, the comparative analysis of these three configurations enables us to clarify the effects of different pore structure parameters on the thermal insulation performance of MJ-fabricated porous ceramics, and further provide targeted structural selection and design references for practical thermal insulation applications, which fully leverages the advantages of MJ technology in fabricating porous ceramics with complex and tailored structures.

### 2.2. Model Development

Modeling of the Octet configuration porous ceramics was completed using SolidWorks 2025 software. A parametric design method was employed to precisely control the lattice unit size, arrangement, and geometric characteristics of the pore structure, ensuring model accuracy and repeatability. Porosity was quantitatively controlled via parametric adjustment of the strut diameter (Octet) and wall thickness (Schwarz, Gyroid) in the modeling software: the target porosity was pre-calculated using the volume ratio method, and the strut/wall dimensions were iteratively adjusted in SolidWorks/MSLattice with real-time volume calculation to achieve the precise porosity values of 40%, 50%, and 60%. The Schwarz and Gyroid configurations were generated using the specialized structural design software MSLattice, which enables the accurate creation of TPMS surfaces. The envelope dimensions of all porous ceramic models were uniformly set to 10 × 10 × 10 mm.

Considering that small wall thicknesses during MJ fabrication of porous ceramics are prone to forming defects (e.g., incomplete sintering, layer delamination) and mechanical damage during post-processing (e.g., cleaning, drying), leading to low forming success rates, the minimum wall thickness of 400 μm was adopted as the design benchmark based on preliminary forming trials and the processing constraints of the MJ equipment, ensuring high fabrication yield and structural integrity of the samples. Finally, a porosity of 60% and a number of periods of 3 were chosen (at this point, the minimum wall thickness of the Octet configuration was 680 μm, the Schwarz configuration was 580 μm, and the Gyroid configuration was 440 μm). Preliminary experiments confirmed Octet and Schwarz as representative high-performance configurations for thermal insulation and structural stability, respectively, and their optimal parameters (60% porosity, 3 periods) were fixed for comparative analysis. Gyroid, as the TPMS configuration with the best comprehensive mechanical performance in preliminary trials, was selected for the construction of additional models with varied porosities (40%, 50%) and period numbers (1, 2) to systematically investigate the independent and synergistic effects of these parameters on thermal insulation performance—this design ensures a targeted yet systematic exploration of all key pore structure parameters in the study. Based on the three basic configurations, additional porous ceramic models with porosities of 50% and 40%, and numbers of periods of 2 and 1 were constructed for the configuration that exhibited superior performance in preliminary experiments, to further analyze the effect of pore structure parameters on thermal insulation.

All designed models were repaired using Materialise Magics 29 software and finally exported as STL files to facilitate subsequent slicing and printing by MJ equipment.

### 2.3. Experimental Work

**Figure 3:**
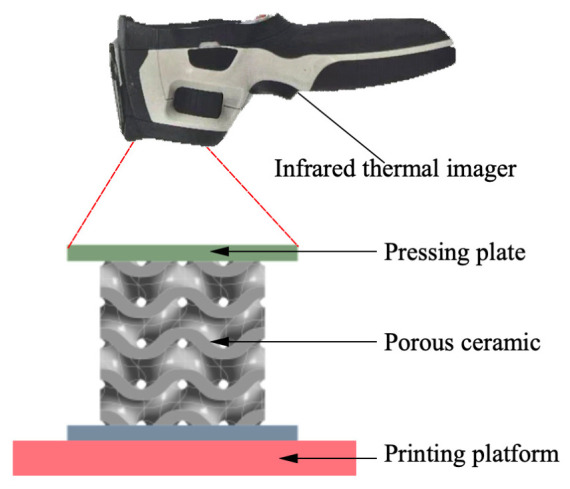
Schematic diagram of the thermal insulation test.

Based on the porous ceramic models with different pore structure parameters, corresponding zirconia porous ceramics were fabricated using the aforementioned MJ equipment and supporting processes, and the related experimental work was demonstrated in Appendix A. Thermal insulation tests were conducted to analyze the effect of pore structure on the thermal performance of porous ceramics. The specific test method is as follows:

After fabricating the MJ zirconia porous ceramic samples, thermal insulation tests were performed as schematically illustrated in Figure 3. For each combination of pore structure parameters, three identical samples were prepared for parallel experiments, and each sample was measured five times to ensure data reliability; the average value of all valid measurements was taken as the final experimental result. The ambient temperature was controlled at 25 °C. The top and bottom pressing plates used in all tests were identical zirconia plates (the same material as the porous ceramics), with a fixed dimension of 10 × 10 × 2 mm (matching the sample’s envelope size, 2 mm in thickness). To minimize contact thermal resistance, all pressing plate surfaces were polished to a roughness of Ra < 0.8 μm to ensure flatness, and a thin layer of thermal conductive silicone was uniformly applied between the plates and the porous ceramic sample for all tests. A pressing plate was placed on both the top and bottom of the porous ceramic sample, which was then placed on the printing platform of the MJ equipment. The printing platform was maintained at a constant temperature of 200 °C to heat the sample. After 100 s, the surface temperature of the upper pressing plate was measured from the top using a CEM DT-980 series handheld infrared thermal imager (Shenzhen Huashengchang Technology Industry Co., Ltd., Shenzhen, China), and the thermal insulation performance was compared. To minimize measurement uncertainties, the emissivity of the upper pressing plate surface was calibrated to 0.95 (the standard emissivity of metal oxide surfaces) for the infrared imager, and thermal conductive silicone was uniformly applied between the porous ceramic sample and the pressing plates to reduce temperature deviations caused by contact resistance. It should be noted that the temperature of the top plate at 100 s is used here as a comparative indicator to evaluate the relative thermal insulation performance of different structures, rather than as a direct measure of intrinsic thermal conductivity.

Further, the infrared thermal imager was operated at a fixed measurement distance of 30 cm and a 90° vertical viewing angle relative to the upper pressing plate surface; the entire surface of the upper pressing plate (10 × 10 mm) was defined as the region-of-interest (ROI) for temperature extraction. The minimum temperature within the ROI was selected as the key thermal insulation performance metric because it directly reflects the ultimate heat transfer inhibition capability of the porous ceramics, which provides a conservative and reliable design basis for practical thermal insulation applications such as lithium-ion battery thermal runaway management—an average ROI temperature would mask the local optimal insulation performance, while the maximum cold-side temperature fails to characterize the core heat-blocking effect of the porous ceramic structure.

Liu et al. [42] experimentally studied the effect of thermal insulation layers on the diffusion of lithium-ion battery thermal runaway. Their results showed that when the battery temperature exceeds 200 °C, thermal runaway is highly likely to occur, and in battery modules without thermal insulation layers, the diffusion rate of thermal runaway is extremely fast-spreading from the first to the fifth battery in only 62 s. A 100 s heating duration was therefore selected to fully capture the thermal insulation performance of the samples under actual battery thermal runaway conditions, as it covers and extends beyond the critical 62 s thermal runaway diffusion time, ensuring the test results reflect the practical protective effect of the porous ceramics for a sufficient duration. Therefore, to make the thermal insulation test more consistent with the actual application scenario, a heating temperature of 200 °C and a heating duration of 100 s were selected.

### 2.4. Numerical Work

In the simulation analysis, three-dimensional finite element models of porous ceramics with different porosities, numbers of periods, and configurations were established based on the ANSYS Workbench platform. By reasonably setting material properties, boundary conditions, and loading methods, the temperature field under high-temperature thermal loads for different pore structure parameters was calculated.

Two materials—zirconia and air—were defined in the model, and their physical properties are listed in Table 1. After material definition, the porous ceramic models of different configurations described in Section 2.2 were imported into ANSYS Workbench, and material assignments were performed for each domain of the models. These parameters are derived from the built-in material library of the ANSYS 2025 software. For simplicity, the thermal conductivities of zirconia and air are assumed to be constant, and these values are applied consistently in all simulations to facilitate comparative analysis.

**Figure 4:**
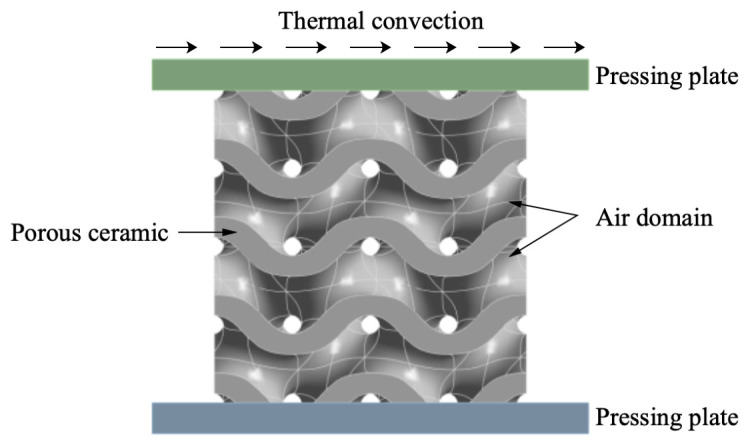
Simulation model and boundary conditions for thermal insulation performance.

After material assignment, boundary conditions were defined with reference to the experimental configuration of the thermal insulation test. Considering the relatively small geometric scale of the porous ceramic structures, the absence of forced convection, and the moderate temperature level (200 °C), natural convection within the pores and thermal radiation were not explicitly included in the simulation model [31,43]. This simplification is based on the understanding that the confined pore size suppresses buoyancy-driven flow, while an order-of-magnitude estimation based on Stefan–Boltzmann linearization suggests that radiative heat transfer is not dominant under the present temperature and geometric conditions. Therefore, these effects are treated as secondary contributions in the current model. As shown in Figure 4, two pressing plates (made of zirconia, consistent with the porous ceramic material) were placed on the top and bottom of the porous ceramic. In addition, the external air domain was represented as a solid region with assigned air thermal properties to account for gas conduction effects. It should be noted that the present simulation does not aim to fully reproduce the experimental boundary conditions, but rather to provide a simplified and consistent framework for comparative analysis of structural effects on thermal insulation performance.

The initial ambient temperature of the simulation model was set to 25 °C. The lower surface of the lower pressing plate was the heating surface, maintained at a constant temperature of 200 °C, while the upper surface of the upper pressing plate was set to natural air convection with a convective heat transfer coefficient of 20 W·(m^2^·K)^−1^. The analysis duration was 100 s, and the thermal insulation performance of porous ceramics was evaluated by comparing the temperature distribution in the simulation model after 100 s. A transient thermal analysis solver was adopted for the simulation, with a fixed time step of 1 s for the 100 s analysis duration. The implicit solution method was used, and the convergence criterion was set to an energy residual of <1 × 10^−6^ to ensure the stability and precision of the temperature field calculation.

Similar to the compressive performance simulation model, tetrahedral meshing was adopted for the thermal insulation simulation model. To improve computational efficiency, the mesh size was set to 0.2 mm for the porous ceramic region and 0.4 mm for the air domain and the two pressing plates. A mesh independence study was conducted to validate mesh size selection: three mesh schemes (0.1 mm/0.2 mm, 0.2 mm/0.4 mm, 0.3 mm/0.6 mm for ceramic/air-plate regions) were tested. The minimum cold-end temperature varied by <3% between the latter two schemes, confirming the 0.2 mm/0.4 mm mesh size balances numerical accuracy and computational efficiency. The quality of each thermal insulation simulation mesh was evaluated using element quality and aspect ratio. Element quality directly reflects the geometric deformation of the mesh; low element quality may lead to increased local errors or even computational divergence, seriously affecting the reliability of thermal analysis results. Aspect ratio, as introduced in the compressive performance simulation model, may cause abnormal elongation or distortion of the heat flow path of mesh elements in thermal conduction analysis if excessively large, resulting in inaccurate calculation of temperature gradients and thus affecting simulation authenticity. The mesh quality of thermal insulation simulations for different pore structures is listed in Table 2.

## 3. Results and Discussion

### 3.1. Experimental Analysis of Thermal Insulation Performance

**Figure 5:**
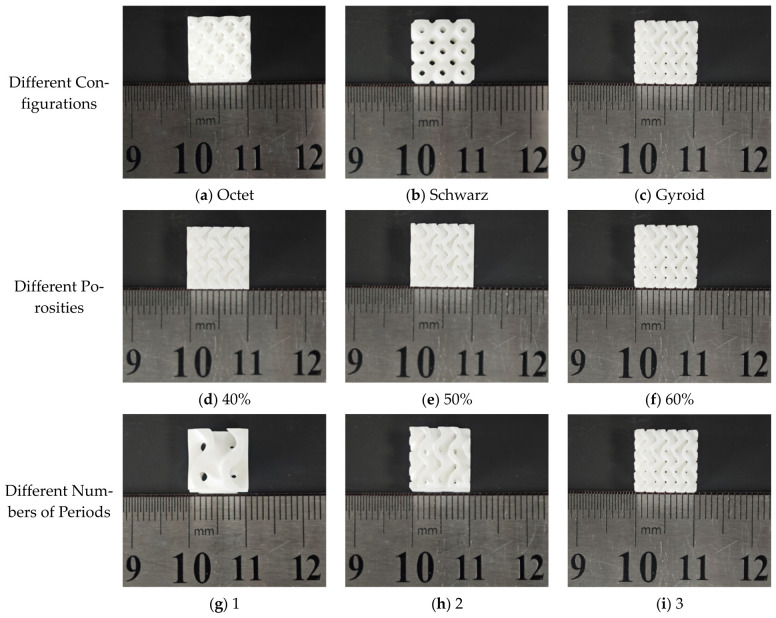
Prepared porous ceramics.

Zirconia porous ceramics with the three configurations (Octet, Schwarz, and Gyroid) described in Section 2.2 were successfully fabricated using MJ equipment and supporting processes. The physical images of the fabricated porous ceramics with different configurations, porosities, and numbers of periods are shown in Figure 5. Thermal insulation tests were conducted in accordance with the method described in Section 2.3.

**Figure 6:**
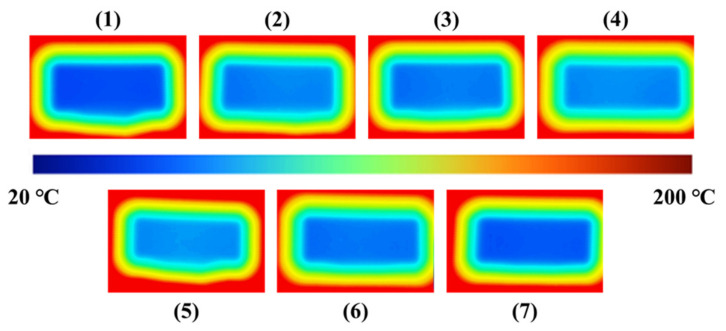
Temperature contour maps of porous ceramics with different pore structures in thermal insulation tests.

The Gyroid configuration was selected to test the thermal insulation performance of porous ceramics with different porosities and numbers of periods. Figure 6 shows the temperature contour maps of the upper pressing plate after heating the porous ceramics on a 200 °C printing platform for 100 s, and Table 3 lists the corresponding minimum temperatures from Figure 6, where the numbers in Figure 6 correspond one-to-one to the groups of porous ceramics with different pore structures in Table 3. It should be acknowledged that the present thermal evaluation approach is not a standardized measurement of thermal conductivity, and the results are primarily intended for qualitative comparison and trend analysis under consistent experimental conditions.

The compressive bearing performance and thermal insulation performance of MJ-fabricated porous ceramics exhibit significant differences with changes in pore structure. As observed from the data in Table 3, the Octet configuration zirconia porous ceramics with 60% porosity and 3 periods exhibit the optimal thermal insulation performance, with the lowest upper pressing plate temperature of 58.5 °C in the thermal insulation test, which is significantly lower than that of other configurations. In contrast, although the Gyroid configuration porous ceramics have the best compressive bearing performance, their thermal insulation performance is inferior to that of the Octet configuration under non-forced convection conditions, with the minimum upper pressing plate temperature reaching 66.3 °C.

Further analysis was conducted on the Gyroid configuration’s thermal insulation performance at different porosities (3 periods fixed). The thermal insulation capacity of Gyroid porous ceramics decreases significantly as porosity reduces. The minimum upper pressing plate temperature rises from 66.3 °C (60% porosity) to 70.5 °C (40% porosity). This trend is the complete opposite of the compressive performance, which improves with decreasing porosity. With porosity fixed at 60%, the Gyroid configuration’s thermal insulation performance enhances as the number of periods decreases. When the period number is reduced to 1, the minimum upper pressing plate temperature is only 60.8 °C, indicating good thermal insulation performance. However, this sample has poor compressive strength, which is significantly lower than that of porous ceramics with a higher number of periods.

### 3.2. Numerical Analysis of the Effect of Pore Structure on Thermal Insulation Performance

#### 3.2.1. Effect of Configuration on Thermal Insulation Performance of Porous Ceramics

**Figure 7:**
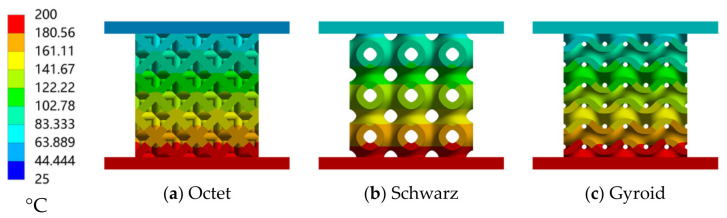
Temperature contour plots of porous ceramics with different configurations.

Thermal insulation simulations of porous ceramics with different configurations were carried out based on the aforementioned simulation model. Table 4 and Figure 7 show the thermal insulation simulation results and temperature contour maps of the Octet, Schwarz, and Gyroid configurations with 60% porosity and 3 periods, respectively. After heating the lower surface of the lower pressing plate at 200 °C for 100 s, a distinct temperature gradient formed at the porous ceramic region can be observed. The minimum cold-end temperature of porous ceramics obtained by simulation is relatively consistent with the experimental results in terms of both value and trend. The porous ceramics with the Octet configuration have the lowest cold-end temperature of 59.2 °C, which is significantly lower than that of the Schwarz and Gyroid configurations, indicating the optimal thermal insulation performance among the three configurations under the same conditions. In contrast, the Schwarz and Gyroid configurations (both TPMS structures) have relatively close minimum cold-end temperatures.

TPMS structures are widely used due to their excellent thermal insulation performance. However, in the thermal insulation tests and simulation models, the Schwarz and Gyroid configurations (both TPMS structures) exhibit slightly inferior thermal insulation performance compared to the Octet configuration. This is mainly because the excellent thermal insulation performance of TPMS structures relies on their complex internal channels, which facilitate heat exchange with gases or liquids to achieve thermal insulation. However, no forced convection was applied to the porous ceramics in the experiments and simulations, and the small forming size resulted in insignificant natural convection inside the pores. As described in the thermal insulation principle of porous ceramics, the effects of natural convection and thermal radiation during heat transfer were ignored. Under non-forced convection conditions, the main heat transfer modes of porous ceramics are solid conduction and gas conduction. Referring to Table 1, the thermal conductivity of zirconia is significantly higher than that of air, so solid conduction contributes much more to the heat transfer of porous ceramics than gas conduction.

**Figure 8:**
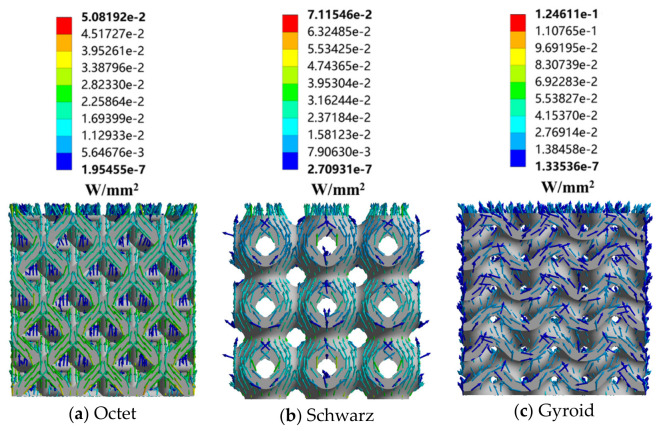
Heat flux vector diagrams of porous ceramics with different configurations.

Figure 8 shows the heat flux vector diagrams in the solid domains of the three configurations. It can be clearly observed that although the continuous curved surface structures of the Schwarz and Gyroid configurations (Figure 8b,c) extend the heat transfer path to a certain extent, they are still inferior to the Octet configuration composed of interlaced strut elements. The tortuous and winding heat conduction path of the Octet configuration (Figure 8a) effectively reduces the overall thermal conductivity of the material, thereby exhibiting superior thermal insulation performance.

#### 3.2.2. Effect of Porosity on Thermal Insulation Performance

**Figure 9:**
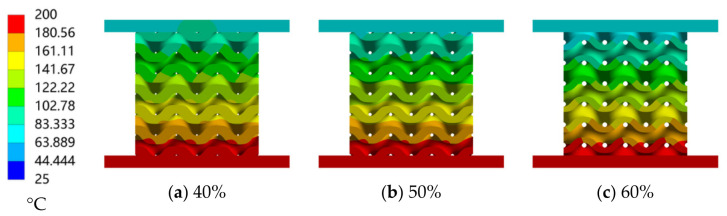
Temperature contour plots of porous ceramics with different porosities.

Figure 9 and Table 5 show the thermal insulation simulation results of the Gyroid configuration porous ceramics with porosities of 40%, 50%, and 60%, respectively. The data indicate that under the same conditions, the thermal insulation capacity of porous ceramics increases significantly with increasing porosity, which is consistent with the experimental results. Due to the inability to completely match the boundary conditions and material parameters in the simulation with the actual experimental conditions, the simulation results are more sensitive to changes in porosity than the experimental results.

In the simulation results, when the porosity decreases from 60% to 50% and 40%, the minimum cold-end temperature of the porous ceramics increases from 65.9 °C to 71.9 °C and 77.8 °C, respectively, with temperature increases of 6.0 °C (60% to 50%) and 6.3 °C (50% to 40%). In the thermal insulation tests, the changes in the minimum cold-end temperature were only 2.6 °C (60% to 50%) and 1.6 °C (50% to 40%).

Observing the changes in the minimum cold-end temperature of porous ceramics with porosity in both experimental and simulation results, it is found that the minimum cold-end temperature in both cases exhibits a nearly linear change with porosity. This is consistent with the thermal conductivity of the solid part of porous ceramics based on porosity and solid material properties described in the thermal insulation principle of porous ceramics. In the principle, the sum of the ratio of the apparent density of porous ceramics to the true density of the solid material and the porosity is 1. When the porosity decreases equally, the ratio increases equally, thereby making the overall thermal conductivity of porous ceramics increase nearly equally. Intuitively, this is reflected in the nearly equal rate of increase in the minimum cold-end temperature of porous ceramics as shown in Table 5, indicating that the effect of porosity on the thermal insulation performance of porous ceramics is relatively uniform.

#### 3.2.3. Effect of Number of Periods on Thermal Insulation Performance

**Figure 10:**
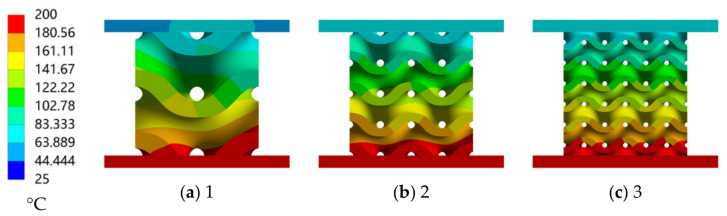
Temperature contour plots of porous ceramics with different periods.

Figure 10 shows the temperature contour maps of the thermal insulation simulations of porous ceramics with different numbers of periods under the same conditions, and Table 6 lists the minimum cold-end temperatures from Figure 10. The data in Table 6 clearly indicate that the simulation results are in good agreement with the experimental results: for the Gyroid configuration, the thermal insulation performance of porous ceramics improves with decreasing number of periods.

Although it is generally believed that a decrease in the number of periods will effectively reduce the heat transfer path in the solid material, thereby reducing the thermal insulation performance of porous ceramics, the Gyroid configuration porous ceramics in the thermal insulation tests and simulation models exhibit the opposite trend.

Analysis of the temperature field distribution characteristics of porous ceramics with different numbers of periods using the simulation model reveals that this phenomenon is related to the structural inhomogeneity of the Gyroid configuration. Observing the temperature contour map of the porous ceramics with 3 periods (Figure 10c), it can be seen that the temperature distribution exhibits a distinct horizontal gradient change. In contrast, the temperature contour maps of the porous ceramics with 2 and 1 periods (Figure 10a,b) show that with decreasing number of periods, the temperature gradient gradually presents a distinct inclined gradient characteristic. This indicates that the inhomogeneous pore distribution of the Gyroid configuration at low numbers of periods forces heat to detour along the solid material structure during transfer. The longer and more tortuous heat conduction path significantly increases the thermal resistance of the porous ceramics and makes the temperature distribution more uneven, thereby resulting in a decrease in the minimum temperature at certain positions of the cold end.

This trend of improved thermal insulation with decreasing number of periods is unique to the Gyroid configuration, arising from its inherent structural inhomogeneity at low period numbers. For the Octet and Schwarz configurations, their highly uniform lattice structures prevent the formation of inhomogeneous pore distributions even with reduced period numbers, and thus this trend is not observed for these two configurations.

## 4. Conclusions

This study investigates the influence of pore structure parameters (configuration, porosity, and number of periods) on the thermal insulation performance of zirconia porous ceramics fabricated by Material Jetting through experiments and numerical simulations. The results show that the Octet configuration, with 60% porosity and 3 periods, exhibits the optimal thermal insulation performance, as its strut-based structure forms a more tortuous heat conduction path, leading to a lower minimum temperature (58.5 °C in experiments and 59.21 °C in simulations) compared to the TPMS-based Schwarz and Gyroid configurations. For the Gyroid configuration, thermal insulation performance improves with increasing porosity—higher porosity reduces the contribution of solid conduction (the dominant heat transfer mode under non-forced convection), while it decreases with decreasing number of periods due to inhomogeneous pore distribution that extends heat transfer paths and enhances thermal resistance. Notably, the thermal insulation trend of porosity is opposite to that of compressive performance, which strengthens with lower porosity. The numerical simulation results based on ANSYS Workbench are consistent with experimental data in both values and trends, verifying the reliability of the simulation model. These findings provide practical references for structural design: the Octet configuration (60% porosity, 3 periods) is recommended for thermal insulation-focused applications like lithium-ion battery thermal runaway management, while the Gyroid configuration with moderate porosity (50–60%) and 2 periods balances thermal insulation and mechanical strength. Overall, this work clarifies the key factors regulating the thermal insulation of MJ-fabricated porous ceramics and offers guidance for their engineering applications.

This study has notable limitations that affect the generalizability of its findings. First, the porous ceramic samples were of a fixed small size, so their thermal insulation behavior may differ for larger engineering-scale samples. Second, only three typical lattice configurations were investigated, with other strut-based or TPMS surface-based structures likely exhibiting distinct thermal insulation performance and heat transfer mechanisms. Third, natural convection and thermal radiation were neglected in numerical simulations, which introduces minor systematic errors and fails to fully reflect heat transfer characteristics in high-temperature or large-pore-volume scenarios. For future research, we will expand the sample size range, test more lattice configurations, and build a comprehensive simulation model that includes natural convection and thermal radiation, to further optimize the structural design of MJ-fabricated porous ceramics for thermal insulation applications.

## Figures and Tables

**Table 1:** Material properties of the numerical model *.

Material	Density	Young’s Modulus	Poisson’s Ratio	Thermal Conductivity	Specific Heat Capacity at Constant Pressure
Zirconia	6.050 × 10^3^ kg·m^−3^	220 GPa	0.3	2.0 W·(m·K)^−1^	400 J·(kg·K)^−1^
Air	1.161 kg·m^−3^	-	-	2.6 × 10^−2^ W·(m·K)^−1^	1007 J·(kg·K)^−1^

* These parameters are from the built-in material library of ANSYS software.

**Table 2:** Mesh quality for the simulation of thermal insulation performance in different porous structures.

Group	Configuration	Porosity	Number of Periods	Average Element Quality	Average Aspect Ratio
1	Octet	60%	3	0.8273 ± 0.1240	1.8765 ± 0.5066
2	Schwarz	60%	3	0.8279 ± 0.0992	1.8715 ± 0.4561
3	Gyroid	60%	3	0.8251 ± 0.1083	1.8846 ± 0.5608
4	Gyroid	50%	3	0.8221 ± 0.1047	1.8873 ± 0.5311
5	Gyroid	40%	3	0.8242 ± 0.1061	1.8778 ± 0.5399
6	Gyroid	60%	2	0.8416 ± 0.0953	1.8278 ± 0.4543
7	Gyroid	60%	1	0.8498 ± 0.0908	1.7944 ± 0.4233

**Table 3:** Minimum temperature in thermal insulation tests of porous ceramics with different pore structures.

Group	Configuration	Porosity	Number of Periods	Minimum Temperature (°C)
1	Octet	60%	3	58.5 ± 0.3
2	Schwarz	60%	3	66.7 ± 0.5
3	Gyroid	60%	3	66.3 ± 0.6
4	Gyroid	50%	3	68.9 ± 0.2
5	Gyroid	40%	3	70.5 ± 0.2
6	Gyroid	60%	2	65.2 ± 0.6
7	Gyroid	60%	1	60.8 ± 0.7

**Table 4:** Minimum temperature in thermal insulation simulations of porous ceramics with different pore structures.

Group	Configuration	Experimental Minimum Temperature (°C)	Simulated Minimum Temperature (°C)
1	Octet	58.5	59.2
2	Schwarz	66.7	66.1
3	Gyroid	66.3	65.9

**Table 5:** Thermal insulation simulation results of Gyroid-structured porous ceramics with 3 periods and different porosities.

Group	Porosity	Experimental Minimum Temperature (°C)	Simulated Minimum Temperature (°C)
1	60%	66.3 °C	65.9 °C
2	50%	68.9 °C	71.9 °C
3	40%	70.5 °C	77.8 °C

**Table 6:** Thermal insulation simulation results of Gyroid-structured porous ceramics with 60% porosity and different numbers of periods.

Group	Number of Periods	Experimental Minimum Temperature (°C)	Simulated Minimum Temperature (°C)
1	3	66.3 °C	65.8 °C
2	2	65.2 °C	64.3 °C
3	1	60.8 °C	58.7 °C

**Figure A1:**
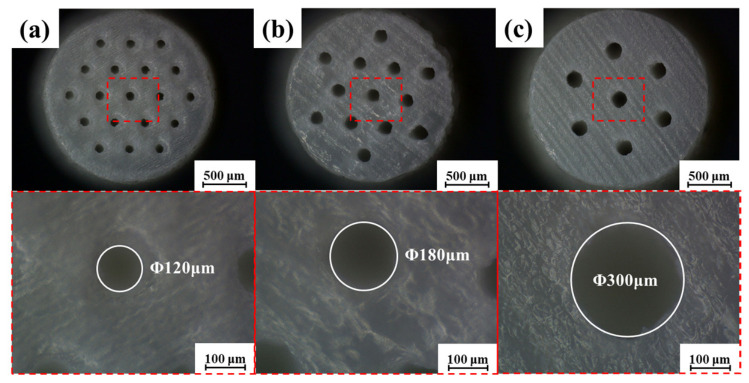
Molding accuracy of circular holes with different diameters: (**a**) target diameter 150 μm (**b**) target diameter 200 μm (**c**) target diameter 300 μm.

## Data Availability

The data presented in this study are available on request from the corresponding author.

## Appendix A

### Appendix A.1

The basic dimensions of the specimens for forming accuracy testing are 36 mm × 3 mm × 4 mm (length × width × height). According to the layout of specimens before printing, three placement angles (0°, 45°, 90°) and two arrangement directions (horizontal and vertical) are adopted. The three-axis forming dimensions of the samples were measured using a digital vernier caliper with an accuracy of 0.01 mm. During measurement, five samples were selected from each group and the average values were calculated. The test results are summarized in Table A1.

**Table A1:** Forming Accuracy of Printed Specimens under Different Spatial Orientations.

Group	Placement Angle	Arrangement Direction	Green Body Dimensions (mm)			Final Sintered Dimensions (mm)			Shrinkage (%)		
			Length	Width	Height	Length	Width	Height	Length	Width	Height
1	0°	Vertical	43.7	3.68	4.89	35.98	3.03	4.01	17.66	17.69	18.05
2	45°	Vertical	43.69	3.69	4.88	35.95	3.03	4.02	17.73	18.02	17.58
3	90°	Vertical	43.68	3.66	4.9	35.96	3.01	4.01	17.67	17.69	18.17
4	0°	Horizontal	43.72	3.68	4.89	35.95	3.01	4.03	17.77	18.14	17.63
